# Efficacy of a topical formulation containing eprinomectin, esafoxolaner and praziquantel (NexGard^®^ Combo) in the treatment of natural respiratory capillariosis of cats[Fn FN1]

**DOI:** 10.1051/parasite/2024005

**Published:** 2024-02-04

**Authors:** Angela Di Cesare, Giulia Morganti, Massimo Vignoli, Mariasole Colombo, Fabrizia Veronesi, Antonello Bufalari, Eric Tielemans, Frederic Beugnet, Donato Traversa

**Affiliations:** 1 Department of Veterinary Medicine, University of Teramo, Località Piano D'Accio snc 64100 Teramo Italy; 2 Department of Veterinary Medicine, University of Perugia Via San Costanzo 4 06126 Perugia Italy; 3 Boehringer Ingelheim Animal Health 29 Avenue Tony Garnier 69007 Lyon France

**Keywords:** *Eucoleus aerophilus*, *Capillaria aerophila*, Cats, Pulmonary capillariosis, Eprinomectin, Treatment

## Abstract

Feline pulmonary capillariosis is a significant disorder due to its distribution and clinical impact. This study evaluated the safety and efficacy of two administrations 28 days apart of a topical solution containing esafoxolaner, eprinomectin and praziquantel (NexGard^**®**^ Combo) in treating *Eucoleus aerophilus* (syn. *Capillaria aerophila*) infection in naturally infected cats. Cats were allocated to two groups: G1 cats (*n* = 23) received two treatments at study days (SDs) 0 and 28 (±2) and were evaluated for 6 weeks, and G2 cats (*n* = 17) served as a negative control for 6 weeks and were then treated twice on SDs 42 (±2) and 70 (±2), allowing for an additional 6-week assessment of efficacy. Each cat was subjected to McMaster coproscopy at SDs −7/0, 28 (±2) and 42 (±2) for both groups, 70 (±2) and 84 (±2) only for G2. Clinical examination and chest radiographic images were performed at SDs 0, 28 (±2) and 42 (±2) for G1 and G2, 70 (±2) and 84 (±2) only for G2. The comparison of EPG (eggs per gram of feces), clinical (CS), and radiographic scores (RS) at each time-point was used as a criterion. The efficacy based on the EPG reduction was 99.5% (G1) and 100% (G2) after two administrations of NexGard^**®**^ Combo 2 weeks apart. At SD 0, no significant differences for CS and RS were recorded between G1 and G2, while a significant reduction (*p* < 0.05) was observed post-treatment for CS, RS, oculo-nasal discharge, auscultation noises, and cough. Two doses of NexGard^**®**^ Combo 28 days apart stopped egg shedding and significantly improved clinical alterations in cats infected by *E. aerophilus*.

## Abstract (fr)

La capillariose pulmonaire féline est un trouble important, de par sa répartition et son impact clinique. Cette étude a évalué l’innocuité et l’efficacité de deux administrations à 28 jours d’intervalle d’une solution topique contenant de l’esafoxolaner, de l’éprinomectine et du praziquantel (NexGard^®^ Combo) dans le traitement de l’infection à *Eucoleus aerophilus* (syn. *Capillaria aerophila*) chez des chats naturellement infectés. Les chats ont été répartis en deux groupes : les chats G1 (*n* = 23) ont reçu deux traitements aux jours d’étude (JE) 0 et 28 (±2) et ont été évalués pendant 6 semaines et les chats G2 (*n* = 17) ont servi de contrôle négatif pendant 6 semaines, puis ont été traités deux fois aux JE 42 (±2) et 70 (±2), permettant une évaluation supplémentaire de l’efficacité sur 6 semaines. Chaque chat a été soumis à une coproscopie McMaster aux JE −7/0, 28 (±2) et 42 (±2) pour les deux groupes, 70 (±2) et 84 (±2) uniquement pour G2. L’examen clinique et les images radiographiques thoraciques ont été réalisés aux JE 0, 28 (±2) et 42 (±2) pour G1 et G2, 70 (±2) et 84 (±2) uniquement pour G2. La comparaison des nombres d’œufs par gramme de matières fécales (OPG), score clinique (SC) et score radiographique (SR) à chaque point ont été utilisées comme critères d’efficacité. L’efficacité basée sur la réduction de OPG était de 99,5 % (G1) et de 100 % (G2) après deux administrations de NexGard Combo à deux semaines d’intervalle. À JE 0, aucune différence significative pour SC et SR n’a été enregistrée entre G1 et G2, tandis qu’une réduction significative (*p* < 0,05) a été observée après le traitement pour SC, SR, écoulements oculo-nasaux, bruits d’auscultation et toux. Deux doses de NexGard^®^ Combo à 28 jours d’intervalle arrêtent l’excrétion des œufs et améliorent considérablement les altérations cliniques chez les chats infectés par *E. aerophilus*.

## Introduction

The trichuroid nematode *Eucoleus aerophilus* (Creplin, 1839) (syn. *Capillaria aerophila*) affects the respiratory tract of different animals, including dogs, cats, and wild carnivores, and occasionally humans [8–11]. The adult stages live beneath the epithelium of trachea, bronchi, and bronchioles and the females lay non-embryonated eggs which are passively transported up the respiratory tract, are coughed up and released in the environment through nasal discharges, or swallowed *via* the pharynx, and eventually released into the environment through feces. Larvae develop inside the eggs reaching the infectious stage, though the life cycle of *E. aerophilus* is yet to be fully elucidated. Cats (and other animals) acquire the infection possibly by ingesting larvated eggs from the environment [1, 2, 10]. The feline infection caused by *E. aerophilus* is often underestimated in clinical practice, despite the broad parasite distribution in Europe, *e.g*., the Mediterranean Basin, Eastern regions, and northern countries [3, 6, 16]. This parasitic nematode causes damage to the pulmonary parenchyma and triggers local inflammation in the respiratory tract. Infections in cats can be subclinical or characterized by chronic bronchitis with signs of varying severity like sneezing, wheezing, and bronchovesicular sounds; most often cats show a dry cough which becomes moist and productive when secondary bacterial infections occur [13–15, 18]. Death is rare but can occur due to severe bronchopneumonia and respiratory failure induced by heavy parasitic burdens [13].

Information on effective and safe anthelmintic treatments of pulmonary capillariosis in cats is patchy and mainly comes from a few case reports. To date, only topical formulations containing the macrocyclic lactone moxidectin 1% w/v (plus the neonicotinoid imidacloprid 10% w/v) are licensed to treat respiratory capillariosis in cats. Additionally, there is evidence that the macrocyclic lactone eprinomectin also has the potential to treat *Eucoleus* spp. infections in cats, including *E. aerophilus* [7]. Given the advantage in expanding treatment options for the infection caused by *E. aerophilus* in feline animals, the present study evaluated the efficacy and safety of a topical product containing eprinomectin (plus esafoxolaner and praziquantel) in the therapy of respiratory capillariosis in naturally infected cats.

## Materials and methods

### Study design

A multi-site, negative controlled, blinded clinical efficacy field study was conducted from January 2022 to June 2023 in three veterinary practices located in endemic regions of central Italy. The study was approved beforehand by the regulatory authority (Italian Health Ministry, Authorization No. 0018513-02/08/2021-DGSAF-MDS-A) and by the Ethics Committee of the Department of Veterinary Medicine of the University of Teramo (2021-UNTECLE-0023140 22/09/2021). All cats were included in the study with written informed consent from the owner. The study relied on a randomized block design based on order of presentation with a targeted 1:1 ratio Investigational Veterinary Product (IVP): Control-IVP (C-IVP), *i.e.*, Group 1 (G1) and Group 2 (G2), respectively. Overall, 41 cats (18 males and 23 females) aged 4 months to 10 years were included in the study. Forty cats completed the study, while one cat from G2 was lost to follow-up after study day (SD) 0. Twenty-three and 17 cats were assigned to G1 (receiving NexGard^®^ Combo at SD 0 and 28 (±2)) or G2 (negative control until SD 42 (±2), then receiving NexGard^®^ Combo at SD 42 (±2) and 70 (±2)), respectively.

Each cat was administered NexGard^®^ Combo per label recommendations.

On SDs −7/0, 28 (±2), and 42 (±2), and only for G2 on SDs 70 (±2) and 84 (±2) cats were evaluated for respiratory capillariosis by quantitative copromicroscopy. Clinical examinations and radiographic imaging were also performed at SDs 0, 28 (±2), and 42 (±2) for both G1 and G2 and at SDs 70 (±2) and 84 (±2) for G2.

After inclusion, animals were strictly monitored by the investigators. Procedures for removal were in place to prevent poor animal health or welfare during the study.

### Pre-inclusion screening and enrolment

Two consecutive fecal samples collected from all cats identified for inclusion were subjected to copromicroscopic analysis between SDs −7 and 0. Samples were examined by quantitative evaluation of *E. aerophilus* egg excretion by determining the eggs per gram of feces (EPG) with a modified McMaster technique. Cats that excreted *E. aerophilus* eggs at least at one of the two pre-inclusion analyses were considered suitable for enrolment and further evaluated for inclusion and exclusion criteria. Additionally, cats had to fulfill the following inclusion criteria to be enrolled in the study: i) ≥0.8 kg of body weight, ii) ≥8 weeks old, iii) healthy (except for *E. aerophilus*-related clinical signs), and iv) positive for *E. aerophilus* eggs at SD −7 to 0. Animals were excluded if they were: i) debilitated or diagnosed with a disease or injury impacting the study, ii) uncooperative or otherwise unsuitable for inclusion, iii) pregnant females or intended for breeding during the study, and iv) treated with a systemic nematodicide within the last 30 days prior to the study start. After inclusion, cats were divided into two groups, *i.e*., G1 or G2.

### Methods and statistical analysis

#### Copromicroscopy

Fecal samples from two different consecutive defecations were obtained for each collection time point and examined by the McMaster technique with a sensitivity of 50 EPG. All individual samples were examined in two replicates for a total of four replicate samples observed from the two consecutive fecal collections. The McMaster method was carried out as previously described using a 1.350 specific gravity zinc sulphate solution [12]. Eggs of *E. aerophilus* were identified by their typical morphometric and morphological features, *i.e.*, size, absence of a space between the embryo and the wall, asymmetry of bipolar plugs, and characteristics of the outer shell [17].

#### Clinical evaluation

At each visit, presence or absence of non-specific and/or respiratory clinical signs compatible with respiratory capillarioses (*e.g*., general respiratory distress, bronchovesicular sounds, sneezing, wheezing, chronic dry cough, and dyspnea) was evaluated and a clinical score was assigned to each cat based on the severity of the clinical pictures (0, 1 or 2 points were assigned for each clinical sign). Total clinical scores (CS) for each cat were calculated by adding the scores for food intake, activity, rectal temperature, cough, mucous membranes, oculo-nasal discharge, respiratory rate, and auscultation (0 corresponding to a normal health condition; the higher the numerical value, the more serious the clinical condition of the animal). Arithmetic means were used for the analysis. After treatment, cats were clinically evaluated for the onset of adverse reactions.

#### Radiographic imaging

Each cat was subjected to chest radiography at each SDs (as described in Study design). The presence and the severity of bronchial, alveolar, reticular interstitial, and nodular interstitial patterns were evaluated, and a radiographic score was assigned to each cat. Specifically, 0, 1, 2, or 3 points were assigned for bronchial, alveolar, and nodular interstitial radiographic alterations, while for reticular interstitial alterations 0 or 1 point were given. Total radiographic scores (RS) for each cat were calculated by adding the radiographic scores of each alteration detected (0 corresponding to an absence of impairment; the higher the numerical value, the more serious the pulmonary alterations of the animal). Arithmetic means were used for the analysis.

#### Statistical analysis

The primary efficacy variable for the efficacy assessment was the comparison of the EPG values on SDs 28 (±2) and 42 (±2) between G1 and G2.

*Eucoleus aerophilus* egg count reduction (%) was calculated for each time point with the formula: 100 × [(arithmetic mean among control G2 − arithmetic mean among the G1 treated animals)/arithmetic mean among control G2].

The treatment efficacy for G2 on SDs 70 (±2) and 84 (±2) was then compared to the SD 42 (±2) baseline values for the G2 group.

The significance of the egg count reduction was analyzed by Wilcoxon non-parametric sum rank test, and all testing was two-sided at the significance level *α* = 0.05.

The proportion of cats negative for *E. aerophilus* eggs in G1 compared to the Control G2 on SDs 28 (±2) and 42 (±2) was analyzed using the Wilcoxon non-parametric sum rank test. Cats were considered negative if no *E. aerophilus* eggs were seen at the McMaster in the two replicates from the two consecutive fecal collections.

Collateral efficacy parameters were the clinical evaluations and the analysis of radiographic scores pre- and post-treatment. Clinical scores were also compared between groups using Wilcoxon non-parametric sum rank tests.

## Results

During the study, none of the cats received concomitant treatments nor showed adverse reactions following drug administration.

### Parasitological efficacy

The efficacy based on the reduction of EPG counts in G1 compared to G2 was 88.33% (*p* < 0.0001) on SD 28 (±2) and 99.52% (*p* < 0.0001) on SD 42 (±2). Efficacy based on the reduction of EPG counts in G2 compared to its baseline was 95.20% (*p* < 0.0001) at SD 70 (±2) and 100% (*p* < 0.0001) at SD 84 (±2) (Table 1). Out of the 23 cats included in G1, 17 (73.9%) and 22 (95.7%) were negative for *E. aerophilus* eggs at SDs 28 (±2) and 42 (±2), respectively. Cats in G2 were persistently infected until day 42 (±2). Fourteen (82.4%) G2 cats were negative for *E. aerophilus* eggs at the first post-treatment evaluation on SD 70 (±2), and all (17) G2 cats were free from the infection at SD 84 (±2). Table 1 shows the number of cats infected by *E. aerophilus* at each time point for both study groups.

**Table 1:** Number of cats infected with *Eucoleus aerophilus* from Group 1 (G1) and Group 2 (G2) and results of quantitative McMaster (EPG counts) at each time point.

Study day	Number of cats infected		Average EPG		% Efficacy G2 as control	% Efficacy G2 Day 42 as baseline
	G1	G2	G1	G2		
−7/0	23/23	18/18	125.5	120.6	NA	NA
28 (±2)	6/23	17/17*	14.7	125.7	88.3	
42 (±2)	1/23	17/17	0.5	114.0	99.5	
70 (±2)	–	3/17		5.5		95.2
84 (±2)	–	0/17		0.00		100.0

*One cat did not complete the study and was lost to follow-up from SD 28 onwards.

### Clinical scores

No significant differences were observed between G1 and G2 at SD 0. In G2, the clinical signs remained consistent during the no-treatment period (SD 0 to SD 42 (±2)). On SD 0, 14/23 (60.9%) cats in G1 and 12/17 (70.6%) cats in G2 presented clinical signs compatible with the infection. Most common signs on SD 0 were alterations on lung auscultation (increased breath sounds, inspiratory or expiratory wheezes or crackles) and dry cough, followed by sneezing and respiratory distress (Table 2). Seven (30.4%) and 4 (17.4%) cats in G1 showed clinical signs at SDs 28 (±2) and 42 (±2), respectively. Ten (58.8%) and 4 (17.4%) cats in G2 had clinical signs at SDs 70 (±2) and 84 (±2), respectively (Table 2).

**Table 2:** Clinical signs and clinical scores of cats from G1 and G2 at each time point.

Clinical sign	Group	Study day				
		0 *n*/tot	28 (±2) *n*/tot	42 (±2) *n*/tot	70 (±2) *n*/tot	84 (±2) *n*/tot
General respiratory distress	1	2/23	0/23	0/23	–	–
	2	2/17	2/17	2/17	0/17	0/17
Sneezing	1	2/23	1/23	0/23	–	–
	2	4/17	4/17	4/17	1/17	0/17
Wheezing	1	0/23	0/23	0/23	–	–
	2	0/17	0/17	1/17	0/17	0/17
Cough	1	7/23	2/23	0/23	–	–
	2	5/17	6/17	6/17	0/17	0/17
Food intake – reduction	1	4/23	1/23	1/23	–	–
	2	3/17	4/17	4/17	1/17	0/17
Mild to moderate lethargy	1	2/23	1/23	0/23	–	–
	2	3/17	2/17	3/17	2/17	1/17
Temperature	1	1/23	0/23	0/23	–	–
	2	0/17	0/17	0/17	0/17	0/17
Pale mucous membranes	1	2/23	1/23	0/23	–	–
	2	1/17	1/17	0/17	0/17	0/17
Ocular-nasal discharge	1	3/23	2/23	1/23	–	–
	2	3/17	6/17	7/17	4/17	3/17
Respiratory rate	1	2/23	1/23	1/23	–	–
	2	5/17	3/17	2/17	1/17	0/17
Respiratory movements	1	1/23	1/23	1/23	–	–
	2	2/17	2/17	2/17	0/17	0/17
Auscultation	1	11/23	2/23	1/23	–	–
	2	7/17	7/17	7/17	4/17	1/17
No. of symptomatic cats	1	14/23	7/23	4/23	–	–
	2	12/17	14/17	14/17	10/17	4/17
Average total clinical score	1	1.4	0.5	0.2	–	–
	2	1.9	1.9	1.9	0.7	0.2

In both G1 and G2, the conditions significantly improved after the first treatment (SD 28 (±2) for G1 and SD 70 (±2) for G2) and almost complete clinical resolution was observed after the second treatment (SD 42 (±2) for G1 and SD 84 (±2) for G2).

### Radiographic scores

At SD −7/0, 20/23 (86.9%) cats in G1 and 16/17 (94.1%) cats in G2 showed radiographic alterations compatible with parasitic pneumonia. On SD 42 (±2), abnormalities of the chest radiography were still present in 17 (73.9%) cats in G1. At SD 84 (±2) radiographic alterations were reported in 11/17 (64.7%) G2-cats (Table 3). Differences for the average total radiographic scores were significant (Table 4).

**Table 3:** Radiographic alterations and radiographic scores of cats from Group 1 (G1) and Group 2 (G2).

Radiographic alteration	Group 1			Group 2				
Study day	0 *n*/tot	28 (±2) *n*/tot	42 (±2) *n*/tot	0 *n*/tot	28 (±2) *n*/tot	42 (±2) *n*/tot	70 (±2) *n*/tot	84 (±2) *n*/tot
Bronchial*								
Total no. of cats	20/23	17/23	15/23	16/17	17/17	17/17	16/17	11/17
Details:								
First generation	5	7	6	3	3	3	8	6
Second generation	8	6	8	7	8	10	6	5
Third generation	7	4	1	6	6	4	2	0
**Alveolar**								
Total no. of cats	5/23	3/23	3/23	0	0	0	0	0
Details:								
Isolated fluffy infiltrates	2	2	2	0	0	0	0	0
Well defined with air bronchograms	3	1	1	0	0	0	0	0
**Reticular interstitial**								
Total no. of cats	14/23	12/23	7/23	10/17	10/17	8/17	5/17	1/17
Details:								
Interstitial framework visible but could be bronchial pattern	5	9	4	7	6	4	4	1
Interstitial framework can be distinguished from bronchial pattern	9	3	3	3	4	4	1	0
**Nodular interstitial**								
Total no. of cats	2/23	0/23	1/23	0/17	0/17	0/17	0/17	0/17
**Total number of cats showing radiographic alterations**	20/23	17/23	15/23	16/17	17/17	17/17	16/17	11/17
**Average total radiographic score**	3.3	2.2	1.7	2.8	3	2.8	1.9	1.0

*Bronchial alterations were defined as mild (i.e., first generation of bronchi visible), moderate (second generation of bronchi visible) and severe (third generation of bronchi visible).

**Table 4:** Average total clinical scores (in particular related to oculo-nasal discharge, lung auscultation, and cough), and average radiographic scores of Group 1 (G1) and Group 2 (G2) at each time point.

Study day	Clinical scores								Radiographic scores	
	Total		Oculo-nasal discharge		Auscultation		Cough		Total	
	G1	G2	G1	G2	G1	G2	G1	G2	G1	G2
0	1.435	1.765	0.130	0.235	0.478	0.353	0.304	0.294	3.261	2.765
28 (±2)	0.478	1.882	0.087	0.412	0.087	0.353	0.087	0.353	2.217	3.000
42 (±2)	0.217	1.882	0.043	0.471	0.043	0.353	0.000	0.353	1.652	2.765
70 (±2)		0.706		0.235		0.235		0.000		1.882
84 (±2)		0.294		0.176		0.059		0.000		1.000

### Clinical efficacy

Median clinical and radiographic scores of G1 and G2 at each time point are shown in Table 4. Significant differences were observed for the mean clinical score and in particular for three clinical parameters (*i.e*., oculo-nasal discharge, auscultation noises, and cough).

The non-parametric Mann-Whitney test (=Wilcoxon Sum Rank Test) demonstrated that the distribution of clinical and radiographic total score was significantly different after treatment; with no differences observed between groups before treatments (Table 5).

**Table 5:** Statistical analysis of clinical and radiographic scores.

Study day	Mean clinical scores				Mean radiographic scores
	Total	Oculo-nasal discharge score	Auscultation score	Cough score	
	*p*-value				
G1/G2					
0	0.47	0.659	0.443	0.958	0.44
G1/G2					
28 (±2)	0.002 (*)	0.039 (*)	0.042 (*)	0.042 (*)	0.11
G1/G2					
42 (±2)	3.09 × 10^−5^ (*)	0.0046 (*)	0.013 (*)	0.0024 (*)	0.01 (*)
G2/G2					
28 (±2)/42 (±2)	0.94 (#)	0.762 (#)	1 (#)	1 (#)	0.51 (#)
G2/G2					
42 (±2)/70 (±2)	0.02 (*§)	0.254	0.472 (°)	0.0084 (*)	0.02 (*§)
G2/G2					
42 (±2)/84 (±2)	0.00041 (*)	0.129	0.0389 (*)	0.0084 (*)	7.33 × 10^−5^ (*)

*Significant difference is considered with *p*-value < 0.05; (#) No change before treatment; (§) start of change with treatment; (°) No change at T1.

## Discussion

The results of the present multi-center clinical field study demonstrated that two topical applications of esafoxolaner/eprinomectin/praziquantel (NexGard^®^ Combo) 28 days apart are both safe and highly effective in treating cats naturally infected with *E. aerophilus*.

The efficacy of this anti-parasitic formulation was defined by analyzing copromicroscopic results, as previously carried out for similar efficacy studies on respiratory nematodes of companion animals [5, 7]. Specifically, the quantitative McMaster technique was performed to assess the infection status before and after treatment. Two administrations of NexGard^®^ Combo two weeks after the second treatment provided efficacy between 99.5% and 100%.

Most treated cats showed improvement in clinical signs and there was a notable decrease of the mean clinical scores within 2 weeks after the second treatment, confirming the high clinical efficacy of NexGard^®^ Combo, as demonstrated by the lower fecal egg count and clinical recovery of the cats. The persistence of clinical signs after treatment in some cats that were negative at McMaster egg counting might be due to significant chronic lesions, which may necessitate more time for full recovery, as hypothesized in previous studies [4]. This hypothesis is also supported by the observation that most study animals exhibited persistent lung lesions at the chest radiographic images 2 weeks after the second treatment, despite the absence of egg shedding.

While it is possible that the sensitivity of fecal examinations may not reach 100%, especially when compared to necropsy, this technique is still the most reliable approach to diagnose respiratory parasitosis in practical settings. The test sensitivity was increased through two consecutive quantitative analyses during both pre- and post-treatment time points to ensure diagnostic accuracy and the reliability of the collected data.

The present study adds to current knowledge on the management of feline capillariosis in clinical practice. The occurrence of respiratory clinical signs in cats with compatible history and signalment, underlines the need to evaluate at-risk animals with fecal floatation and Baermann test [13, 15, 18]. In fact, clinical pictures of feline tracheobronchial eucoleosis overlap with those caused by aelurostrongylosis and troglostrongylosis, for which the Baermann test represents the gold standard diagnostic technique.

Based on the present findings, the following protocol for the treatment and monitoring of feline pulmonary capillariosis is suggested. All infected cats should undergo a comprehensive physical examination, including chest radiographic images. Following the diagnosis and the completion of the treatment regimen (consisting of two administrations 28 days apart), a recommended schedule would entail monthly check-ups until the animal achieves full recovery (*i.e*., when clinical signs and radiographic abnormalities disappear and the fecal floatation test shows negative results).

In conclusion, this multi-center clinical field trial demonstrated that NexGard^®^ Combo is a safe and highly efficacious treatment for *E. aerophilus* infection in cats under field conditions. The findings of this study contribute to the growing body of evidence supporting the use of NexGard^®^ Combo as a reliable solution for managing parasitic infections in feline populations [19–21].

## Conflict of Interest

This study was financed by Boehringer Ingelheim Animal Health, of which Eric Tielemans and Frederic Beugnet are employees.
